# Integrating phosphoproteomics into the clinical management of prostate cancer

**DOI:** 10.1186/s40169-017-0138-5

**Published:** 2017-02-14

**Authors:** Larry C. Cheng, Victor M. Tan, Shridar Ganesan, Justin M. Drake

**Affiliations:** 1 Rutgers Cancer Institute of New Jersey, New Brunswick, NJ 08901 USA; 2 Graduate Program in Cellular and Molecular Pharmacology, Graduate School of Biomedical Sciences, Rutgers, The State University of New Jersey, Piscataway, NJ 08854 USA; 3 Graduate Program in Quantitative Biomedicine, Graduate School-New Brunswick, Rutgers, The State University of New Jersey, Piscataway, NJ 08854 USA; 4 Division of Medical Oncology, Department of Medicine, Rutgers Robert Wood Johnson Medical School, New Brunswick, NJ 08901 USA

**Keywords:** Biomarkers, Clinical diagnostics, Clinical trial design, Kinase inhibitors, Pharmacotherapy, Phosphoproteomics, Precision medicine, Prostate cancer, Mass spectrometry

## Abstract

Phosphoproteomic analysis of tumor samples has the potential to uncover significant insights into kinase signaling networks present in late stage prostate cancer that are complementary to genomic and transcriptomic approaches. Phosphoproteomics could potentially aid drug development in clinical trial design as well as provide utility for oncologists in the personalized therapeutic management of individual cancers through identifying novel biomarkers and druggable targets. Rapid advancement of targeted mass spectrometry platforms is underway to integrate phosphoproteomic technology with genomic assays to soon translate this information into the cancer clinic.

## Current clinical state of prostate cancer

In 2016, the American Cancer Society estimates 181,890 new cases of prostate cancer in men in the United States, which ranks as the highest among cancers in men and second only to lung cancer in estimated deaths at 26,120 [1]. In advanced stages of the disease, androgen deprivation therapy (ADT) is the primary approach to reduce tumor burden. This hormonal therapy is effective initially, as assessed by the decline in prostate specific antigen (PSA) levels. However, this response is not durable and almost always results in relapse, termed castration resistant prostate cancer (CRPC). CRPC is often accompanied by the development of radiographically visible metastases. Metastatic castration-resistant prostate cancer (mCRPC) represents the lethal form of the disease where prognosis is poor with a median survival time of less than two years [2]. The best available treatment options for mCRPC patients, including second-generation antiandrogens and taxane-based chemotherapies, modestly improve survival by a few months [2–4]. This highlights the urgent need to identify more impactful agents and a patient-specific approach tailored to treat each tumor uniquely.

Unlike in early stage disease, where the development of the PSA test to screen patients for prostate cancer has both raised awareness and improved survival [1], finding useful biomarkers for patient stratification or therapy in mCRPC remains a clinical challenge [3, 4]. One issue is that prostate cancer has relatively low mutation rates when compared to other types of cancer [5, 6]. Nonetheless, several fascinating papers that analyzed the genomic and transcriptomic landscape of primary prostate cancer and mCRPC identified numerous point mutations, translocations, and amplifications [5–11]. While several of these mutations may be deemed actionable, no effective treatments have yet been developed that target a majority of these mutations, with the exception of DNA repair and androgen receptor (AR) mutations. Indeed, a recent clinical trial illustrated that patients with BRCA2 or ATM functional loss (deletion or mutation) responded favorably to olaparib whereas patients without these mutations did not [12]. Another breakthrough paper found that patients who exhibited resistance to abiraterone acetate or enzalutamide harbor the AR-V7 splice variant in their prostate circulating tumor cells (CTCs) [13]. These selected examples of novel biomarkers will no doubt improve the treatment landscape for patients with mCRPC. However, due to modest improvements in overall survival, it is important to consider other strategies such as proteomics and/or phosphoproteomics to illuminate our understanding of the pathways that drive this disease. These methods would complement nicely with existing genomic and transcriptomic information to prioritize the key drivers and corresponding treatments in mCRPC.

## Integrating clinical omics

Since the turn of the century, genomic technologies have matured to a point where practicality is no longer in question. Advances in sequencing technologies have both reduced cost and increased effectiveness of obtaining genomic and transcriptomic data. In parallel, advances in the biomedical field has benefited from the increase in knowledge from sequencing. Projects such as The Cancer Genome Atlas (TCGA) and International Cancer Genome Consortium (ICGC) have provided a rich resource of genomic information for scientists to apply to their research. As a result, clinical practice has gradually transformed to incorporate genomics in diagnosis, patient stratification, and treatment selection [14]. Conversely, clinical proteomic and phosphoproteomic platforms have taken longer to develop, owing to the greater complexity and difficulty involved with analyzing proteins or phosphoproteins in very small tissue amounts (i.e., biopsy).

Even with these current limitations proteomics and phosphoproteomics have provided a wealth of information from clinically relevant cancer tissues. Work by us and the National Cancer Institute’s Clinical Proteomic Tumor Analysis Consortium (CPTAC; previously the Clinical Proteomic Technology Assessment for Cancer) have published some groundbreaking papers in colorectal, ovarian, breast, and prostate cancer characterizing the genomic, proteomic, and/or phosphoproteomic landscapes in these diseases [15–19]. These studies have provided clues into the signaling networks related to each cancer type through incorporation of novel proteomic and phosphoproteomic data. Importantly, these studies have opened up new opportunities to develop and test computational strategies that will integrate proteomic or phosphoproteomic information with existing genomic data. Hence, a significant next step will be to extract clinically actionable information from these integrated approaches.

In prostate cancer, previous work by our group on the tyrosine phosphoproteome in mCRPC patients identified several activated tyrosine kinases and observed interpatient heterogeneity but similarity among metastatic sites within the same patient [19]. This may imply that evaluation of the phosphoproteome of a singly biopsy could reveal the crucial activated signaling pathways to inform treatment decisions or aid in the development of prognostic or predictive biomarkers during response or relapse to current therapies in mCRPC patients. The main issue, though, was devising a method to portray which kinases or pathways to prioritize from within the complex phosphoproteomic data. Our recent publication in *Cell* [16] attempted to resolve this issue through a defined, systematic approach that integrated several omic datasets from mCRPC patient tumor samples using a novel computational pipeline [20]. Our analysis revealed that inclusion of the phosphoproteomic data provided more functional pathway information after integration with genomics and transcriptomics versus the integration of only genomics and transcriptomics. Certain pathways (e.g., AKT/mTOR/MAPK, nuclear receptor, and cell cycle signaling) were found to be significantly enriched in mCRPC when the phosphoproteomic data was included but only marginally enriched when excluded [16]. To easily visualize specific patients’ signaling networks in the context of canonical cancer hallmark pathways, we created the phosphorylation-based cancer hallmarks using integrated personalized signatures (pCHIPS). It is important to note that every patient we evaluated had enrichment of at least four cancer hallmarks making prioritization of kinase pathways still very challenging.

To overcome this, we incorporated the pathway information to procure a set of targetable kinases predicted to have maximal effect on these cancer hallmarks. These patient-specific kinase hierarchies make it possible to stratify mCRPC patients according to these hierarchies using targeted kinase inhibitors, in combination with other agents, for maximum therapeutic potential [16]. Two major findings came from this approach: (1) Not every patient would be predicted to respond to the same kinase inhibitor (even though the same cancer hallmark pathway may be enriched) and (2) Involvement of the cell cycle pathway is very prominent in a majority of these patient samples suggesting that CDK4/6 kinase inhibitors may be highly efficacious clinically, especially in combination with other targeted agents. It should be mentioned that our phosphoproteomic data could also be influenced by activation (or repression) of phosphatases. Our focus was on activated kinases, but we cannot rule out the notion that hyperphosphorylation of these kinases (or their substrates) may be regulated by phosphatase activity. In addition to kinase inhibitors, our phosphoproteomic information could also help inform the selection of phosphatase inhibitors, which may provide a greater pleiotropic effect when compared to single agent kinase inhibitors, though none are currently FDA-approved [21]. While these results are exciting, further experimental validation in vitro and in vivo is needed to confirm the implicated pathways.

## Clinical phosphoproteomics: opportunities and challenges

**Fig. 1:**
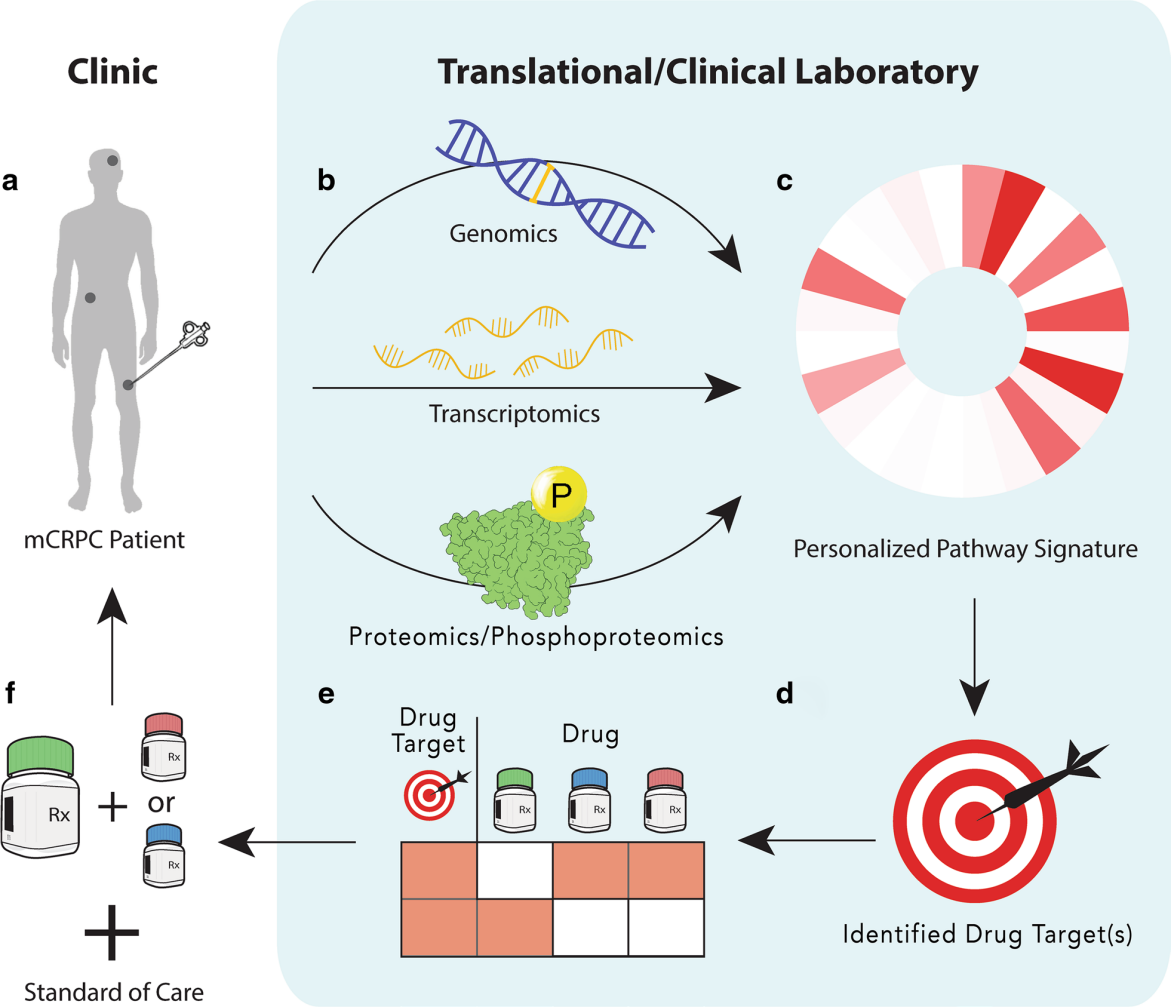
Conceptual diagram of the integration of phosphoproteomics into prostate cancer clinical management. **a** A biopsy of the tumor is taken from a metastatic castration-resistant prostate cancer (mCRPC) patient. **b** The biopsy specimen is processed to generate its omics data. **c** The omics data is integrated to produce a personalized pathway signature. **d** Drug targets are identified from this signature. **e** Available drugs are assessed for each drug target. **f** The drug or combinations of drugs that block the target are selected for treatment in addition to standard of care as an arm in a clinical trial

Kinase inhibitors have long been important players in the area of targeted therapies against cancer. However, clinical trials using kinase inhibitors where randomization is preferred over biomarker stratification have reported mixed results, with a majority of these studies not demonstrating any clinical benefit, especially in prostate cancer [22, 23]. An explanation for some of these clinical trial failures may result from primary or acquired drug resistance mechanisms. Many resistance pathways have been discovered during the administration of therapies targeting driver kinase mutations in several cancers and hence can reduce the durability of these targeted agents [24, 25]. For example, it has been observed that EGFR pathway activity is responsible for the continued growth and survival of BRAF (V600E) colon cancers resistant to vemurafenib, an observation not observed in BRAF (V600E) melanomas [26]. It is our view that future clinical trial successes will hinge on properly stratifying patients according to predicted drug response by utilizing biomarkers that accurately reflect the tumor’s biology. The incorporation of phosphoproteomics with genomics and transcriptomics could help characterize how a patient’s tumor responds to treatment as well as tease out any future mechanisms of resistance providing these necessary biomarkers. In that light, a soon to be initiated phase II clinical trial in prostate cancer (NCT03012321) will utilize genomics to pre-select patients with DNA repair mutations followed by randomization to evaluate the efficacy of olaparib alone or in combination with an androgen synthesis inhibitor, abiraterone acetate. As we move forward, it would be expected that biomarker-driven clinical trials will be the norm rather than the exception, providing clinicians with a method for stratifying late stage disease as well as selecting the appropriate therapy for each stratum (Fig. 1).

Phosphoproteomic technologies have made significant progress in the past decade with several platforms available: antibody-based assays (e.g., reverse phase protein arrays) and MS-based assays. MS-based assays are further delineated into discovery/global and targeted approaches [27]. Antibody-based approaches require low sample amounts, which makes them practical in the clinical setting where biopsies are typically performed. Furthermore, these assays do not require special instrumentation, easing their adoption into clinical laboratories. However, this approach is reliant on the availability of quality, specific antibodies, which can be more difficult when evaluating phosphorylation. MS-based approaches can bypass this limitation and perform multiplexed analysis of many proteins or phosphoproteins at once [27]. MS-based assays, specifically the targeted platform, carry great potential for future clinical utility especially in the space of diagnostic and predictive biomarker development. The global MS platform allows for relative quantitative comparisons of an unknown list of phosphopeptides between samples. The technique involves extracting proteins from the sample and digesting with a protease (usually trypsin plus Lys-C) into peptides and phosphopeptides [28]. Due to the relatively lower abundance of phosphoproteins, the phosphopeptides require enrichment from the complex mixture [29]. This can be done using immobilized metal affinity chromatography (IMAC) [30] such as TiO_2_ or antibodies (for phosphotyrosine enrichment) [31]. The enriched sample is then separated by liquid chromatography and detected via tandem MS. Detected peptides and phosphopeptides can be analyzed using software programs such as MaxQuant [32] or Skyline [33]. However, global MS is limited by its reproducibility due to the biased nature of the MS method [34]. Systemic bias in data collection arises from the complex digests and experimental design [34, 35]. Furthermore, phosphopeptides of higher abundance are sampled more frequently and precisely while phosphopeptides of lower abundance may be missed, creating a missing values problem [34, 35]. Efforts are ongoing in standardizing normalization strategies and data imputation methods to address these challenges both within experiments and across experiments [35, 36]. Targeted MS can bypass these limitations by measuring specifically annotated peptides that are constituent to a protein of interest. By using isotope-labeled standards and acquiring MS data specifically for the peptides of interest, targeted MS can precisely and reproducibly quantitate that protein in the sample by using previously mentioned programs such as Skyline [34]. Adoption of targeted MS to examine biomedical problems is growing and best practices are emerging to begin to standardize the field [37]. Crucial to this effort, the CPTAC network of laboratories demonstrated high reproducibility of this approach within and between laboratories as well as across instruments in measuring protein concentrations in plasma [38].

As phosphoproteomic technologies mature we foresee a paradigm shift. Clinicians will order tests that evaluate pathway activity as well as mutational status and will apply these results in real-time in the clinic. Until that day arrives, more work is still needed in the field of phosphoproteomics that improve upon some of the same sets of challenges that early work in the genomics field faced nearly 15 years ago. Phosphoproteomic MS platforms require greater amounts of sample as well as greater upfront investment on infrastructure in terms of equipment and operator expertise [27]. Furthermore, the assay workflow is lengthy, limiting reproducibility as well as practicality. We anticipate that many of these issues will be solved with continual technological advancement. Reducing the sample requirement amount will make the platform more practical for biopsy-based tests in the clinic. Indeed, a recent landmark paper demonstrated reproducible detection and quantification of over 2000 proteins from 18 clinical biopsy samples by utilizing novel pressure cycling technology and sequential window acquisition of all theoretical fragment ion mass spectra (SWATH-MS) [39]. While exciting, further investigation is necessary to determine if phosphopeptides can be detected in the same manner due to the relatively lower abundance of phosphopeptides when compared to total peptides. Efforts to simplify the workflow [40] will reduce the turn-around time between sample collection and results, enabling clinicians to more quickly evaluate and determine the next step in disease management.

## Conclusions

While MS-based targeted phosphoproteomics is in preclinical stages of research and development, we believe that the eventual translation of this technology will open new doors in the clinical setting. Phosphoproteomics, as an integrative approach with genomics and other omics data, may have a future hand in addressing the challenges of prostate cancer (and other cancer) diagnoses and drug development by identifying actionable pathways. The technology would also pave the way for a more comprehensive field of pharmaco-omics to rationally select and modify a patient’s drug therapy for different diseases that have low mutation burden.

## Abbreviations

ADT: androgen deprivation therapy
AR: androgen receptor
CPTAC: Clinical Proteomic Tumor Analysis Consortium
CTC: circulating tumor cell
ICGC: International Cancer Genome Consortium
IMAC: immobilized metal affinity chromatography
mCRPC: metastatic castration-resistant prostate cancer
MS: mass spectrometry
pCHIPS: Phosphorylation-based Cancer Hallmarks using Integrated Personalized Signatures
PSA: prostate-specific antigen
SWATH-MS: sequential window acquisition of all theoretical fragment ion mass spectra
TCGA: The Cancer Genome Atlas
